# Antimicrobial-Resistant Bacteria in Infected Wounds, Ghana, 2014^1^

**DOI:** 10.3201/eid2405.171506

**Published:** 2018-05

**Authors:** Hauke Janssen, Iryna Janssen, Paul Cooper, Clemens Kainyah, Theresia Pellio, Michael Quintel, Mathieu Monnheimer, Uwe Groß, Marco H. Schulze

**Affiliations:** University Medical Center Goettingen, Goettingen, Germany (H. Janssen, I. Janssen, M. Quintel, M. Monnheimer, U. Groß, M.H. Schulze);; St. Martin de Porres Hospital, Eikwe, Ghana (P. Cooper, C. Kainyah, T. Pellio)

**Keywords:** wound infections, Ghana, rural, gram-negative bacteria, Enterobacteriaceae, polymicrobial infections, resistance, AMR, MRSA, multiresistant, antimicrobial resistance, bacteria

## Abstract

Wound infections are an emerging medical problem worldwide, frequently neglected in under-resourced countries. Bacterial culture and antimicrobial drug resistance testing of infected wounds in patients in a rural hospital in Ghana identified no methicillin-resistant *Staphylococcus aureus* or carbapenem-resistant *Enterobacteriaceae* but identified high combined resistance of *Enterobacteriaceae* against third-generation cephalosporins and fluoroquinolones.

Bacteriologic investigation of clinical specimens is an essential tool for active surveillance of antimicrobial drug resistance. Knowledge of causative bacterial species and their resistance profile enables targeted antimicrobial therapy, limits ineffective antimicrobial therapy, and avoids in part unnecessary antimicrobial pressure to noninvolved bacterial pathogens (*1*). Available antimicrobial resistance data will sensitize clinicians and policy makers and are a prerequisite for updating national treatment guidelines (*1*,*2*). These data contribute to prevention and control of antimicrobial drug resistance (*1*).

Wound infections are an emerging medical problem worldwide; the economic burden and morbidity and mortality rates are huge (*3*,*4*). Because of the frequent polymicrobial nature of infected wounds, bacteriologic investigations are demanding and frequently neglected in sub-Saharan Africa countries (*5*).

## The Study

Since 2000, the Institute for Medical Microbiology of the University Medical Center Goettingen, Goettingen, Germany, has assisted the running of the bacteriology laboratory in St. Martin de Porres Hospital in Eikwe, Ghana (*2*). Eikwe is a rural coastal village in the Western Region of Ghana; its mission hospital has an admission capacity of ≈200 beds and serves ≈380,000 persons. 

During March–July 2014, we conducted a prospective study at St. Martin de Porres Hospital, performing bacteriologic investigations of infected wounds of inpatients and outpatients during routine working hours (Monday–Friday, 8 am–4 pm). The hospital administration (the local ethics review panel) authorized the study. Patients from whom wound swab samples were investigated provided consent to be included in the study.

Medical doctors diagnosed wound infections clinically, according to the classic signs of inflammation. After wounds were carefully cleaned with sterile gauze moistened with a sterile solution of 0.9% sodium chloride, samples were collected from the wound ground and edge on sterile cotton swabs and immediately transported to the bacteriology laboratory in Amies transport medium (Copan, Brescia, Italy). The samples were inoculated onto MacConkey agar and 7% sheep blood agar (Tulip Diagnostics, Goa, India) and thereafter incubated aerobically at 35°C. Both plates were read after 24 and 48 hours. Gram staining was performed to ensure wound specimen quality and to check for bacteria, neutrophils, and epithelial cells.

Bacterial isolates were initially identified (to genus level) by colony morphology, Gram staining, catalase reaction, oxidase reaction, coagulase reaction, indole reaction, and growth on Kligler iron agar, as described by Cheesbrough (*6*). Bacterial isolates were stored in microbanks at −20°C. Species identification was completed (to species level) at the Institute for Medical Microbiology in Goettingen, Germany, by using MALDI Biotyper 3.0 (Bruker Daltonics, Bremen, Germany).

According to locally available resources, antimicrobial resistance testing was performed through disk diffusion, which guided the treatment of the wound infections. Antimicrobial resistance testing was repeated with VITEK 2 (bioMérieux, Marcy-l’Étoile, France) at the Institute for Medical Microbiology by using AST-P632, AST-P586, AST-N214, and AST-N248 cards with respect to bacterial species and according to the breakpoint tables for interpretation of MICs in EUCAST version 4.0 (*7*). Quality control was performed with the reference strains *Pseudomonas aeruginosa* ATCC 27853, *Escherichia* *coli* ATCC 25922, and *Staphylococcus aureus* ATCC 29213.

Of the 67 wound swab samples, 39 (58.2%) were from female patients. The mean age of the 67 patients was 40.1 ± 20.8 years (range 1–90 years, median 39 years). Of the 67 samples, collection sites were upper extremity for 4 (6.0%), trunk/head for 15 (22.4%), lower extremity for 39 (58.2%), and laparotomy site for 9 (13.4%) (Technical Appendix). A hospital-acquired wound infection was diagnosed for 21 (31.3%) patients. 

All investigated wound swab samples grew bacterial pathogens. Overall, 32 species of bacteria were isolated; median was 3 (range 1–7) species/specimen. Of the 189 isolated species, 72 (38.1%) were *Enterobacteriaceae*, 69 (36.5%) were gram positive, and 48 (25.4%) were nonfermenters (Technical Appendix Table 2). Of the 67 samples, infection was monomicrobial in 17 (25.4%) and polymicrobial in 50 (74.6%). The most frequently detected bacterium in monomicrobial and polymicrobial infections was *S. aureus*. The predominant bacteria in polymicrobial infections were *Enterobacteriaceae* and nonfermenters (Technical Appendix Table 3). Results of VITEK 2 antimicrobial resistance testing of the most frequently found bacterial species are shown in Table 1.

**Table 1:** Percentages of antimicrobial drug resistance in selected bacterial species in wound infections, Ghana, 2014*

Drug	% Resistant							
	*Staphylococcus aureus,* n = 31	*Enterococcus faecalis*, n = 21	*Proteus mirabilis*, n = 20	*Escherichia coli*, n = 19	*Klebsiella pneumoniae*, n = 13	*Enterobacter cloacae* complex, n = 10	*Pseudomonas aeruginosa*, n = 20	*Acinetobacter. baumannii* complex, n = 8
PEN	93.5							
AMP		0	70.0	94.7	100	100		
OXA	0							
SAM		0	45.0	82.2	69.2	100		
TZP			0	10.5	46.2	30.0	10.0	
CXM			5.0	57.9	46.2	80.0		
CTX			5.0	47.4	46.2	40.0		
CAZ			5.0	47.4	46.2	40.0	5.0	37.5†
IPM							0	0
MEM			0	0	0	0	5.0	0
ERY	3.2	100						
CLI	3.2	100						
TET	67.7	100						
GEN	3.2		15.0	46.2	46.2	40.0	10.0	62.5
AMI							0	0
CIP			20.0	46.2	46.2	30.0	15.0	37.5
LVX	0							
SXT	32.3	100	75.0	69.2	69.2	50.0		
FOF	0							
RIF	0							
VAN	0	0						

*Antimicrobial susceptibility testing was performed with VITEK 2 (bioMérieux, Marcy-l'Étoile, France) according to the EUCAST breakpoint tables for interpretation of MICs, version 4.0, 2014 (*7*). Blank cells indicate no testing performed. AMI, amikacin; AMP, ampicillin; CAZ, ceftazidime; CIP, ciprofloxacin; CLI, clindamycin; CTX, cefotaxime; CXM, cefuroxime; ERY, erythromycin; FOF, fosfomycin; GEN, gentamicin; IPM, imipenem; LVX, levofloxacin; MEM, meropenem; OXA, oxacillin; PEN, penicillin; RIF, rifampin; SAM, ampicillin/sulbactam; SXT, trimethoprim/sulfamethoxazole; TET, tetracycline; TZP, piperacillin/tazobactam; VAN, vancomycin. †The interpretation of the CAZ MIC for *A. baumannii* complex followed the recommendations of the Clinical and Laboratory Standards Institute performance standards for antimicrobial susceptibility testing (*8*).

The spectrum of isolated bacteria is comparable to that reported by other studies from sub-Saharan Africa countries, such as Nigeria (*9*), Tanzania (*3*), and Rwanda (*10*). Frequently, studies describe detected pathogens at the genus level only (*3*,*10*). Concerning the proportion of gram-positive to gram-negative pathogens, we isolated slightly more gram-positive pathogens than others (*3*,*9*–*11*).

One of the most common bacteria found in wound infections is *S. aureus* (*3*,*5*,*10*–*12*), which was most frequently identified in our study (Technical Appendix Table 2); however, we detected no methicillin-resistant *S. aureus* (MRSA). In contrast, studies from urban areas in sub-Saharan Africa countries found MRSA rates of >80% (*10*,*12*). Urban areas are centers of specialized healthcare, where many patients who may already have a long medical history are referred. Such referrals predispose urban patients, staff, and others to more MRSA colonization and infection than experienced by those in rural areas (*13*). The hospital in Eikwe is a general hospital; the villagers are mainly fishermen, and there are no big animal farms in the area. Predisposition to MRSA in this area may be low.

We found no carbapenem resistance in *Enterobacteriaceae* (Table 1). Of great concern were the high rates of resistance of *E. coli*, *Klebsiella pneumoniae*, and *Enterobacter cloacae* complex against third-generation cephalosporins, fluoroquinolones, or both (Table 2), as have been found in other studies from urban areas (*3*,*5*,*10*). The indiscriminate use of antimicrobial drugs contributes to this factor (*14*). Officially, selling antibiotics without prescription is not allowed in Ghana; however, almost every oral antimicrobial drug is available over the counter without any prescription. Eikwe is no exception, although the spectrum of available antimicrobial drugs may be smaller there than in cities. Development of antimicrobial drug resistance may also be enhanced by circulation of counterfeit drugs (*15*).

**Table 2:** Ratio of percentages of antimicrobial drug resistance against third-generation cephalosporin CTX and the fluoroquinolone CIP in selected *Enterobacteriaceae* isolated from wound infections, Ghana, 2014*

Drug resistance	% Resistant			
	*Proteus mirabilis*, n = 20	*Eshcerichia* *coli*, n = 19	*Klebsiella pneumoniae*, n = 13	*Enterobacter cloacae* complex, n = 10
CTX-S + CIP-S	80.0	42.1	53.8	60.0
CTX-S + CIP-R	15.0	10.5	ND	ND
CTX-R + CIP-S	ND	5.3	ND	10.0
CTX-R + CIP-R	5.0	42.1	46.2	30.0

*Antimicrobial susceptibility testing was performed by using VITEK 2 (bioMérieux, Marcy-l'Étoile, France) according to the EUCAST breakpoint tables for interpretation of MICs, version 4.0, 2014 (*7*). CIP, ciprofloxacin; CTX, cefotaxime; ND, not detected; R, resistant; S, susceptible.

Resistance of *E. coli* and *K. pneumoniae* against third-generation cephalosporins probably occurs through production of extended spectrum β-lactamase; in *E. cloacae* complex, it is probably through AmpC–β-lactamase. However, this statement is only an assumption because we did not perform molecular analyses.

In Eikwe, rain falls throughout the year and humidity is almost constant at 70%–90% despite 2 rainfall peaks (May–June and October–November). The effect of seasonality on the incidence of wound infections and the frequency of infection with gram-negative bacteria may not be so pronounced as that found in other studies from sub-Saharan Africa countries with high variations in humidity (*9*). However, because we analyzed only swab samples collected during March–July, the effect of seasonality is difficult to evaluate.

## Conclusions

Antimicrobial drug resistance among gram-negative organisms seems to be widespread in Ghana, even among community-onset infections in rural, resource-limited settings, although MRSA was surprisingly absent. Future research efforts should focus on the transmission dynamics and prevention of gram-negative antimicrobial resistance in those settings. Microbiological investigation of the worldwide problem of wound infections should be encouraged in areas of limited resources and might provide a valuable contribution to the surveillance of increasing antimicrobial resistance, especially in *Enterobacteriaceae*, and for the treatment of affected patients.

Technical Appendix Sites and modes of acquisition of wound infections, bacterial species detected in monomicrobial and polymicrobial wound infections, sites of wound infection and detected bacterial species among 67 patients, Ghana, 2014.
