# Supplementary material for: Antimicrobial-Resistant Bacteria in Infected Wounds, Ghana, 2014
Source: Emerg Infect Dis. 2018 May;24(5):916–9. doi: 10.3201/eid2405.171506 (PMC5938797; doi:10.3201/eid2405.171506)
Supplement: Technical Appendix — Sites and modes of acquisition of wound infections, bacterial species detected in monomicrobial and polymicrobial wound infections, sites of wound infection and detected bacterial species among 67 patients, Ghana, 2014. [file 17-1506-Techapp-s1.pdf]

Article DOI: https://doi.org/10.3201/eid2405.171506

Antimicrobial Resistance in Wound Infections, Ghana, 2014

Technical Appendix

Technical Appendix Table 1. Site and mode of acquisition of wound infection in 67 patients, rural Ghana

Localization	No. of patients	Acquisition
upper extremity		
abscess over shoulder	1	community-acquired
abscess of finger	1	community-acquired
infection of palm injury	1	community-acquired
infection of lower arm injury	1	community-acquired
trunk/head		
abscess of cheek	2	community-acquired
abscess of back	2	community-acquired
abscess of abdominal wall	2	community-acquired
ulcerated mastitis	2	community-acquired
ulcerated tumor over scapula	1	community-acquired
infected herniotomy wound epigastric	2	hospital-acquired
infected herniotomy wound inguinal	1	hospital-acquired
syringe abscess of buttocks	2	hospital-acquired
ulcerated scrotum tumor	1	community-acquired
lower extremity		
cellulitis	6	community-acquired
infected skin graft	4	hospital-acquired
infected entry wound of Steinmann-pin	2	hospital-acquired
infected entry wound of external fixator	1	hospital-acquired
infected injury of toe	2	community-acquired
infected injury of foot	3	community-acquired
infected injury of lower leg	7	community-acquired
infected injury of upper leg	4	community-acquired
abscess of upper leg	1	community-acquired
infected ulcer of foot	8	community-acquired
abscess of foot	1	community-acquired
laparotomy wound		
laparotomy wound	9	hospital-acquired

Technical Appendix Table 2. Detected bacterial species in monomicrobial and polymicrobial wound infection of 67 wound swabs

	Monomicrobial wound infection (n = 17)	Polymicrobial wound infection (n = 50)
Detected bacterial species	No. of swabs with bacterial species / total number of swabs (%)	
Gram-positive bacteria		
Staphylococcus aureus	8/17 (47.0%)	23/50 (46.0%)
Enterococcus faecalis		21/50 (42.0%)
Enterococcus faecium		3/50 (6.0%)
Enterococcus gallinarum		3/50 (6.0%)
Corynebacterium striatum		2/50 (4.0%)
Enterococcus avium		2/50 (4.0%)
Enterococcus raffinosus		2/50 (4.0%)
Arthrobacter cuminsii		1/50 (2.0%)
Bacillus cereus		1/50 (2.0%)
Bordetella trematum		1/50 (2.0%)
Corynebacterium freneyi		1/50 (2.0%)
Streptococcus pyogenes		1/50 (2.0%)
Enterobacteriaceae		
Proteus mirabilis	1/17 (5.9%)	19/50 (38.0%)
Escherichia coli	2/17 (11.8%)	17/50 (34.0%)

Detected bacterial species	Monomicrobial wound infection (n = 17)	Polymicrobial wound infection (n = 50)
	No. of swabs with bacterial species / total number of swabs (%)	
Klebsiella pneumoniae		13/50 (26.0%)
Enterobacter cloacae complex	1/17 (5.9%)	9/50 (18.0%)
Proteus vulgaris		2/50 (4.0%)
Providencia stuartii		2/50 (4.0%)
Serratia marcescens		2/50 (4.0%)
Citrobacter freundii		1/50 (2.0%)
Citrobacter koseri	1/17 (5.9%)	
Enterobacter kobei		1/50 (2.0%)
Salmonella Typhi		1/50 (2.0%)
Nonfermenting bacteria		
Pseudomonas aeruginosa	3/17 (17.6%)	17/50 (34.0%)
Acinetobacter baumannii complex	1/17 (5.9%)	7/50 (14.0%)
Kerstersia gyiorum		7/50 (14.0%)
Alcaligenes faecalis		4/50 (8.0%)
Pseudomonas mendocina		3/50 (6.0%)
Achromobacter xylosoxidans		2/50 (4.0%)
Stenotrophomonas maltophilia		2/50 (4.0%)
Myroides species		1/50 (2.0%)
Ochrobactrum intermedium		1/50 (2.0%)

Technical Appendix Table 3. Site of wound infection and detected bacterial species

Upper extremity (n = 4)		Trunk/head (n = 15)		Laparotomy wounds (n = 9)		Lower extremity (n = 39)	
No. of swabs with detected bacterial species from the respective site of wound infection / No. of all swabs from the respective site of wound infection (%)							
E. coli	2/4 (50.0%)	*S. aureus*	7/15 (46.7%)	*E. coli*	6/9 (66.7%)	*S. aureus*	18/39 (46.2%)
S. aureus	2/4 (50.0%)	*P. aeruginosa*	6/15 (40.0%)	*S. aureus*	4/9 (44.4%)	*E. faecalis*	17/39 (43.6%)
A. baumannii c.	1/4 (25.0%)	*E. coli*	4/15 (26.7%)	*E. faecalis*	2/9 (22.2%)	*P. mirabilis*	15/39 (38.5%)
K. pneumoniae	1/4 (25.0%)	*K. pneumoniae*	3/15 (20.0%)	*P. aeruginosa*	2/9 (22.2%)	*P. aeruginosa*	11/39 (28.2%)
P. aeruginosa	1/4 (25.0%)	*P. mirabilis*	3/15 (20.0%)	*A. baumannii* c.	1/9 (11.1%)	*K. pneumoniae*	8/39 (20.5%)
P. mirabilis	1/4 (25.0%)	*A. baumannii* c.	2/15 (13.3%)	*C. striatum*	1/9 (11.1%)	*E. cloacae* c.	7/39 (18.0%)
S. maltophilia	1/4 (25.0%)	*E. cloacae* c.	2/15 (13.3%)	*E. cloacae* c.	1/9 (11.1%)	*E. coli*	7/39 (18.0%)
		E. faecalis	2/15 (13.3%)	*E. kobei*	1/9 (11.1%)	*K. gyiorum*	6/39 (15.4%)
		E. faecium	1/15 (6.7%)	*E. faecium*	1/9 (11.1%)	*A. baumannii* c.	4/39 (10.3%)
		K. gyiorum	1/15 (6.7%)	*K. pneumoniae*	1/9 (11.1%)	*A. faecalis*	4/39 (10.3%)
		P. vulgaris	1/15 (6.7%)	*P. mirabilis*	1/9 (11.1%)	*E. gallinarum*	3/39 (7.7%)
						P. mendocina	3/39 (7.7%)
						A. xylosoxidans	2/39 (5.1%)
						E. avium	2/39 (5.1%)
						E. raffinosus	2/39 (5.1%)
						P. stuartii	2/39 (5.1%)
						S. marcescens	2/39 (5.1%)
						A. cuminsii	1/39 (2.6%)
						B. cereus	1/39 (2.6%)
						B. trematum	1/39 (2.6%)
						C. freundii	1/39 (2.6%)
						C. koseri	1/39 (2.6%)
						C. freneyi	1/39 (2.6%)
						C. striatum	1/39 (2.6%)
						E. faecium	1/39 (2.6%)
						Myroides spp.	1/39 (2.6%)
						O. intermedium	1/39 (2.6%)
						P. vulgaris	1/39 (2.6%)
						S. Typhi	1/39 (2.6%)
						S. maltophilia	1/39 (2.6%)
						S. pyogenes	1/39 (2.6%)

Abbreviations: *A. xylosoxidans*, *Achromobacter xylosoxidans*; *A. baumannii* c., *Acinetobacter baumannii* complex; *A. faecalis*, *Alcaligenes faecalis*; *A. cuminsii*, *Arthrobacter cuminsii*; *B. cereus*, *Bacillus cereus*; *B. trematum*, *Bordetella trematum*; *C. freundii*, *Citrobacter freundii*; *C. koseri*, *Citrobacter koseri*; *C. freneyi*, *Corynebacterium freneyi*; *C. striatum*, *Corynebacterium striatum*; *E. cloacae* c., *Enterobacter cloacae* complex; *E. kobei*, *Enterobacter kobei*; *E. avium*, *Enterococcus avium*; *E. faecalis*, *Enterococcus faecalis*; *E. faecium*, *Enterococcus faecium*; *E. gallinarum*, *Enterococcus gallinarum*; *E. raffinosus*, *Enterococcus raffinosus*; *E. coli*, *Escherichia coli*; *K. gyiorum*, *Kerstersia gyiorum*; *K. pneumonia*, *Klebsiella pneumonia; O. intermedium*, *Ochrobactrum intermedium*; *P. mirabilis*, *Proteus mirabilis*; *P. vulgaris*, *Proteus vulgaris*; *P. stuartii*, *Providencia stuartii*; *P. aeruginosa*, *Pseudomonas aeruginosa*; *P. mendocina*, *Pseudomonas mendocina*; *S. Typhi*, *Salmonella Typhi*; *S. marcescens*, *Serratia marcescens*; *S. aureus*, *Staphylococcus aureus*; *S. maltophilia*, *Stenotrophomonas maltophilia*; *S. pyogenes*, *Streptococcus pyogenes*.
